# A Heterogeneous Phenotypic Presentation of a PALB2 Mutation Carrier With Multiple Metachronous Primary Malignancies: A Case Report

**DOI:** 10.7759/cureus.115554

**Published:** 2026-08-31

**Authors:** Pujita Julakanti, Victor Hugo Spitz, Gerard Runko, Laura Ziton

**Affiliations:** 1 Internal Medicine, Nova Southeastern University Dr. Kiran C. Patel College of Osteopathic Medicine, Fort Lauderdale, USA; 2 Family Medicine, Northwest Medical Center, Margate, USA; 3 Family Medicine, Broward Health, Coral Springs, USA; 4 Family Medicine, Nova Southeastern University Dr. Kiran C. Patel College of Osteopathic Medicine, Fort Lauderdale, USA

**Keywords:** cascade testing, case report, colorectal cancer, genetic testing, germline mutation, hereditary cancer, hereditary cancer syndrome, metachronous malignancies, palb2, pancreatic adenocarcinoma

## Abstract

The Partner and Localizer of BRCA2 (PALB2) gene is a tumor suppressor gene with a role in DNA repair. Pathogenic autosomal dominant mutations of this gene significantly increase lifetime cancer risk, particularly breast and pancreatic cancers. We present a case of a 71-year-old African American woman without a known family history of malignancy found to have a heterozygous PALB2 mutation following the diagnoses of metachronous colorectal adenocarcinomas and multiple gynecologic cancers. After the discovery of the mutation, the patient underwent resection of a pancreatic head adenocarcinoma. Moreover, following an incidental discovery of evolving right-breast microcalcifications on a routine mammogram, the patient is pending a biopsy procedure for further investigation. Her positive mutation results prompted genetic testing in her family, leading to the detection of the mutation in both her daughter and granddaughter.

This case highlights the importance of hereditary cancer evaluation and multigene testing in patients with multiple primary malignancies. While pancreatic cancer is an established PALB2-associated malignancy, the colorectal, uterine, and vulvar cancers observed in this patient should be interpreted cautiously, as their association with PALB2 remains unestablished. Identification of a pathogenic variant can guide risk-appropriate surveillance and cascade testing.

## Introduction

The PALB2 gene is a tumor suppressor gene that plays a crucial role in maintaining genomic stability [1]. It encodes a BRCA2-binding protein that links BRCA1 and BRCA2, forming a genomic surveillance complex that facilitates homologous recombination DNA repair [2]. While homozygous mutations of this gene cause Fanconi anemia by an autosomal recessive mutation, heterozygous carriers have an increased risk of developing breast and pancreatic cancers, with additional cancer associations under investigation [3,4]. Specifically, heterozygous carriers have a 33%-58% lifetime risk of developing breast cancer and a 5-10% lifetime risk of developing pancreatic cancer [4,5].

Although PALB2 pathogenic variants are relatively rare compared to BRCA1/2 variants, their identification rate is increasing due to the use of multigene panel testing [6]. Historically, the mutation was underrecognized due to a greater focus on BRCA genes alone [3,4].

Despite expanding knowledge of PALB2-associated cancer risks, the difficulty in identifying carriers lies in heterogeneous clinical presentations, with some carriers recognized only after the development of malignancy [6]. Penetrance appears variable both between and within families, complicating risk stratification and surveillance strategies [3,4]. The increased cancer risk associated with PALB2 underscores the importance of appropriate genetic evaluation and guideline-directed management. PALB2-deficient tumors have demonstrated sensitivity to platinum-based chemotherapy and poly(ADP-ribose) polymerase (PARP) inhibitors in preclinical and clinical literature [7-9]. Therapeutic relevance, however, depends on the molecular characteristics of an individual tumor and should not be inferred from germline carrier status alone [7-9]. PALB2 identification also informs established breast surveillance recommendations, while pancreatic surveillance is reserved for appropriately selected individuals according to guideline-defined risk criteria [9,10].

This case report describes a heterogeneous clinical presentation in a PALB2 pathogenic variant carrier with multiple metachronous primary malignancies, including pancreatic, gynecologic, and colorectal cancers, as well as an evolving breast abnormality under current surveillance, without a reported family history of malignancy. The occurrence of multiple metachronous primary malignancies across organ systems in a single PALB2 carrier remains relatively underreported [9]. This clinical course illustrates the challenges of recognizing a potential hereditary cancer predisposition in the absence of a suggestive family history or early-onset disease and reinforces the importance of considering comprehensive genetic evaluation in patients with multiple primary malignancies.

## Case presentation

A 71-year-old African American female with a medical history significant for restrictive lung disease, hypertension, and peripheral neuropathy, and no known family history of cancer, presented with an extensive oncologic history involving multiple metachronous primary malignancies. A detailed chronological summary of her malignancies, diagnostic evaluations, and interventions is presented in Table 1, illustrating the progression of multiple metachronous primary cancers across organ systems.

**Table 1:** Chronological timeline of malignancies, diagnostic evaluations, and interventions in a PALB2 mutation carrier

Year (Age)	Malignancy / Finding	Organ System	Diagnostic Modality	Intervention	Outcome / Notes
2012 (58)	Uterine cancer	Gynecologic	Endometrial biopsy	Total hysterectomy	No reported recurrence
2021 (67)	Rectosigmoid adenocarcinoma	Colorectal	Screening colonoscopy + biopsy	Laparoscopic left colectomy	Pathology-confirmed adenocarcinoma
2022 (68)	Right-sided intramucosal adenocarcinoma	Colorectal	Surveillance colonoscopy + biopsy	Laparoscopic-assisted right hemicolectomy	Metachronous primary tumor
Early 2024 (70)	Vulvar cancer	Gynecologic	Clinical evaluation + biopsy	Surgical resection	Local disease treated surgically
Early 2024 (70)	PALB2 pathogenic mutation identified	Genetic	Multigene hereditary cancer panel	Genetic counseling initiated	Cascade testing positive in daughter and granddaughter
Mar 2025 (71)	Right-breast microcalcifications	Breast	Diagnostic mammogram	Surveillance recommended	Initial benign morphology
Jun 2025 (71)	Pancreatic head mass	Pancreas	CT abdomen	Surgical referral	Symptomatic presentation
Jul 2025 (71)	Pancreatic ductal adenocarcinoma	Pancreas	Surgical pathology	Pancreaticoduodenectomy (Whipple)	Adjuvant chemotherapy initiated
Sep 2025 (71)	Evolving breast microcalcifications	Breast	Repeat mammogram + ultrasound	Image-guided biopsy planned	Results pending

As summarized in Table 1, the patient developed sequential primary malignancies, including uterine, colorectal, and vulvar cancers, before identification of a PALB2 mutation.

Identifying the pattern of recurrent and multi-organ malignancy, multigene hereditary cancer panel testing was performed using next-generation sequencing, evaluating high-penetrance cancer susceptibility genes, including *BRCA1*, *BRCA2*, *PALB2*, *ATM*, and *CHEK2*. The patient was found to carry a heterozygous pathogenic PALB2 variant. Cascade genetic testing was subsequently performed in first-degree relatives. The patient’s daughter (age 46) and granddaughter (age 21) were each confirmed to carry the same pathogenic PALB2 variant through targeted testing. Both individuals were referred for genetic counseling and initiation of risk-appropriate surveillance, though neither has been diagnosed with malignancies.

Moreover, in June 2025, she developed abdominal bloating, early satiety, and constipation. The evaluation of these symptoms with an abdominal CT revealed a pancreatic head mass consistent with pancreatic ductal adenocarcinoma. In July 2025, the patient underwent a pancreaticoduodenectomy (Whipple procedure). Postoperative pathology confirmed a malignant pancreatic head neoplasm, prompting the initiation of adjuvant chemotherapy.

She presented to her primary care physician for a postoperative follow-up appointment in August 2025. An examination revealed a well-healed incision with a primary postoperative complaint of intermittent constipation.

Due to her history of malignant neoplasms and high risk of breast cancer associated with PALB2 mutations, she underwent a bilateral diagnostic mammogram in March 2025, which showed right-breast microcalcifications. These findings indicated the need for a follow-up ultrasound, completed in the same month. The ultrasound was negative for signs of malignancy, and a repeat mammogram was recommended in six months. In September 2025, a repeat mammogram revealed changes in the calcification morphology, prompting an ultrasound-guided needle biopsy.

The patient continues to follow up with primary care, oncology, and gastroenterology while breast biopsy results are pending.

Detailed pathologic staging information for each malignancy was not consistently available in the patient’s records. However, the colorectal tumors and the pancreatic adenocarcinoma were managed with surgical resection without documented metastatic disease at the time of diagnosis.

## Discussion

As emerging evidence shows comparable clinical manifestations to BRCA2, recognizing PALB2 as a high-penetrance cancer susceptibility gene is essential. Although PALB2 pathogenic variants have been reported in patients with multiple primary malignancies, this patient’s constellation of five metachronous primary malignancies in the absence of a family history of cancer is unusual. The pancreatic adenocarcinoma is consistent with the established association between PALB2 pathogenic variants and increased pancreatic cancer risk [3,4]. 

PALB2-deficient tumors have shown sensitivity to DNA-damaging therapies, including platinum agents and PARP inhibitors, in the literature [7,8]. However, this patient is known to have a germline PALB2 pathogenic variant, and tumor-specific evidence of biallelic PALB2 loss or homologous recombination deficiency was not available. Accordingly, genotype-directed therapy may represent a potential consideration in an appropriate clinical and molecular context, but no specific treatment recommendation for this patient can be inferred from germline PALB2 status alone.

Moreover, the patient’s breast imaging findings, while not a confirmed malignancy, reflect the importance of guideline-directed breast screening in mutation carriers. Current recommendations for PALB2 carriers include annual MRI and mammography beginning at age 30 due to increased lifetime breast cancer risk [9,11]. The progression of the patient’s breast findings of microcalcifications from benign features in just six months further reinforces the need for vigilant breast surveillance in genetically predisposed patients, such as this patient’s daughter and granddaughter.

The current NCCN Clinical Practice Guidelines in Oncology (NCCN Guidelines®) emphasize risk management for breast, ovarian, pancreatic, and prostate cancer in PALB2 mutation carriers [10,12]. Importantly, pancreatic surveillance is not indicated solely on the basis of PALB2 carrier status in every individual, as eligibility depends on guideline-defined risk criteria, recommending consideration of surveillance for PALB2 carriers with a first- or second-degree relative with exocrine pancreatic cancer from the same side of the family as the pathogenic variant [10]. A comparison between these recommendations and the patient’s clinical course is summarized in Table 2.

**Table 2:** Comparison of NCCN Guidelines® for PALB2 mutation carriers and this patient’s clinical course NCCN: National Comprehensive Cancer Network.

Organ System	NCCN Guidelines Recommendation (PALB2)	Evidence Basis	Patient Outcome	Clinical Implication
Breast	Annual mammography+MRI starting at age 30	High	Surveillance initiated late; evolving microcalcifications at age 71	Earlier identification could have facilitated guideline-directed breast surveillance.
Pancreas	Consider screening in selected individuals at high risk	Limited	Pancreatic adenocarcinoma diagnosed following symptomatic presentation	PALB2 is associated with increased pancreatic cancer risk; however, surveillance eligibility cannot be inferred from PALB2 carrier status alone.
Colon	No formal recommendation	Limited / emerging	Two separate primary colorectal adenocarcinomas	Observed in this patient; a causal relationship with PALB2 is not established.
Gynecologic	Ovarian: Consider risk-reducing salpingo-oophorectomy starting at age 45-50 years	High for ovarian risk management	Uterine and vulvar cancers	Observed in this patient; the relationship of uterine and vulvar cancers to PALB2 remains uncertain.
Family members	Cascade testing recommended	High	Daughter and granddaughter mutation-positive	Preventive opportunity achieved

**Figure 1:**
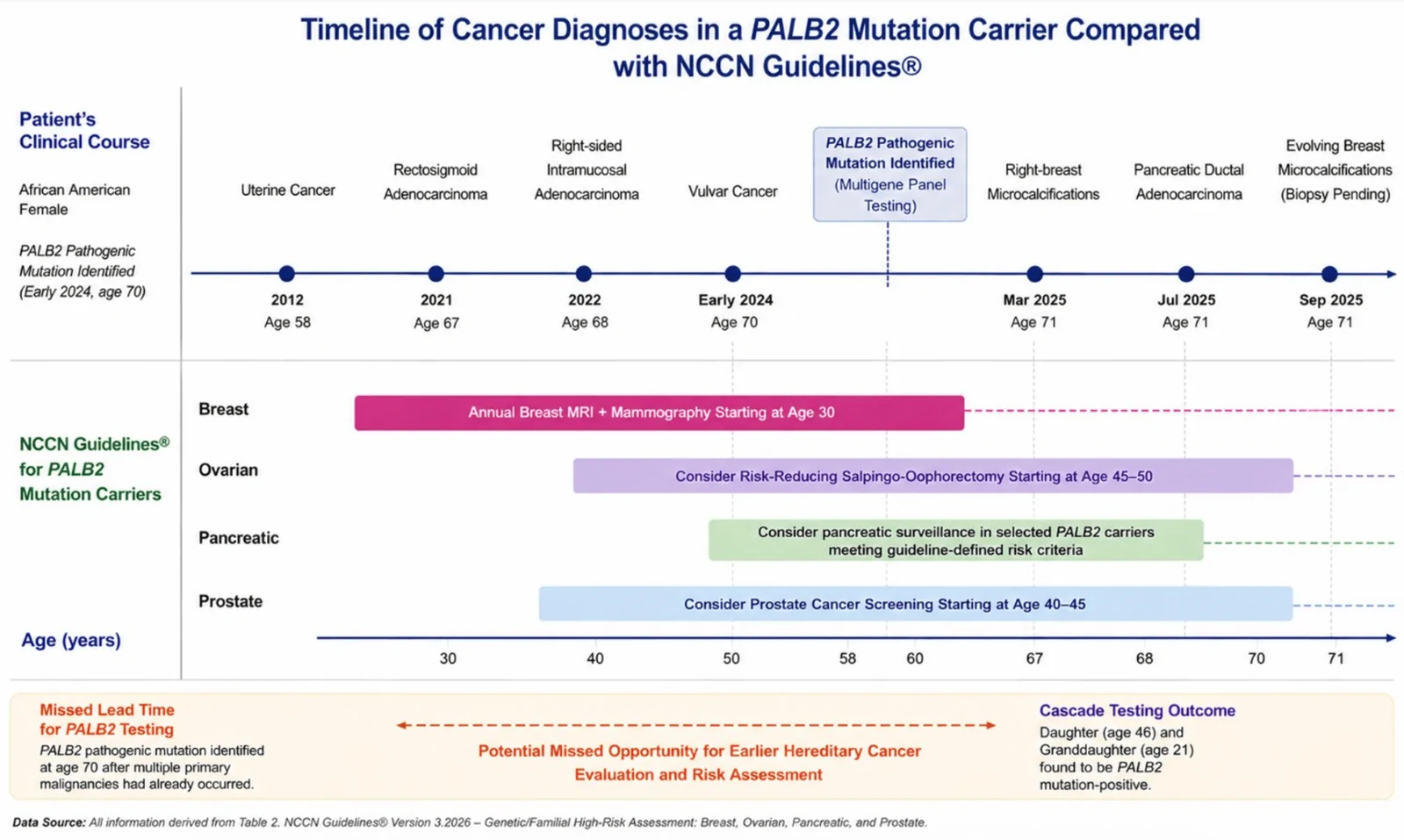
Timeline of cancer diagnoses in a PALB2 mutation carrier compared with NCCN Guidelines screening and genetic testing recommendations This figure presents a chronological visualization of the patient’s oncologic history in relation to NCCN Guidelines for PALB2 mutation carriers and illustrates the timing of genetic testing. The top panel depicts the patient’s clinical course along a horizontal timeline, with corresponding ages at each event. The middle panel summarizes NCCN surveillance and management recommendations for PALB2 mutation carriers by organ system [10], with pancreatic surveillance identified as conditional on guideline-defined risk criteria. The bottom panel highlights the potential missed opportunity for earlier hereditary cancer evaluation and risk assessment. NCCN: National Comprehensive Cancer Network. Figure created using Adobe Illustrator (Adobe, San Jose, CA, USA).

A graphical timeline comparing the patient’s oncologic course with the NCCN Guidelines shown in Table 2 and highlighting the potential missed opportunity for earlier hereditary cancer evaluation and risk assessment is presented in Figure 1 [10].

Taking these recommendations into account, the accumulation of multiple primary malignancies between 2021 and 2024 should have prompted consideration of an underlying hereditary cancer predisposition and referral for genetic evaluation rather than suspicion for PALB2 specifically. Earlier multigene hereditary cancer testing could have identified the PALB2 pathogenic variant sooner and facilitated guideline-directed risk assessment, appropriate breast surveillance, genetic counseling, and earlier cascade testing of at-risk relatives. Although PALB2 is associated with increased pancreatic cancer risk, pancreatic surveillance recommendations are selective. As this patient had no reported family history of pancreatic cancer, she would not have met the family-history criterion that is necessary for pancreatic surveillance in PALB2 carriers.

This patient’s history raises the possibility that patients with PALB2-induced genetic instability may occasionally present with a broader range of malignancies than those currently established. However, existing evidence most clearly supports increased risks of breast and pancreatic cancers, while associations with colorectal and other gynecologic malignancies remain incompletely defined [9,13,14]. Accordingly, the colorectal, uterine, and vulvar cancers observed in this patient are clinically notable, but their occurrence in this patient does not establish a causal association with PALB2. Larger cohort and molecular studies would be required to determine whether such malignancies represent reproducible components of the PALB2-associated cancer spectrum.

Alternative etiologies, including sporadic tumor development, age-related risk accumulation, and environmental exposures, must also be considered. However, the occurrence of multiple metachronous primary malignancies across distinct organ systems within a relatively limited timeframe in this case raises suspicion for an underlying genetic predisposition rather than coincidental processes alone [15].

Cascade genetic testing in this context proved valuable, as it identified two additional mutation-positive family members. Following identification of a pathogenic PALB2 variant, first-degree relatives should undergo genetic counseling and cascade testing to guide risk-appropriate surveillance. In patients with multiple primary malignancies, early-onset cancers, pancreatic cancer, or a suggestive family history, multigene hereditary cancer panel testing should be considered [12]. Early genetic referral remains an important component of care because it can facilitate individualized management and timely cascade testing for at-risk relatives.

Overall, this report illustrates the marked heterogeneity and variable penetrance associated with heterozygous PALB2 mutations and underscores the limitations of relying solely on family history or age at onset when considering genetic testing. Identification of a pathogenic variant can inform guideline-directed risk assessment and surveillance and can create opportunities for cascade testing and preventive care in at-risk relatives.

## Conclusions

This case describes an unusual constellation of multiple metachronous primary malignancies in a carrier of a PALB2 pathogenic variant. The accumulation of multiple primary malignancies should prompt consideration of hereditary cancer evaluation and multigene testing, even in the absence of a reported family history. Identification of a pathogenic variant can guide individualized, guideline-directed management and facilitate cascade testing for at-risk relatives.

## References

[1] Nepomuceno TC, De Gregoriis G, de Oliveira FM, Suarez-Kurtz G, Monteiro AN, Carvalho MA (2017). The role of PALB2 in the DNA damage response and cancer predisposition. Int J Mol Sci.

[2] Redington J, Deveryshetty J, Kanikkannan L, Miller I, Korolev S (2021). Structural insight into the mechanism of PALB2 interaction with MRG15. Genes (Basel).

[3] Hu C, Hart SN, Polley EC (2018). Association between inherited germline mutations in cancer predisposition genes and risk of pancreatic cancer. JAMA.

[4] Yang X, Leslie G, Doroszuk A (2020). Cancer risks associated with germline PALB2 pathogenic variants: an international study of 524 families. J Clin Oncol.

[5] Masanam MK, Pitcher CW, Sogunro O, Starks LK, Murray AB, Boisvert ME (2022). Risk-reducing surgery in PALB2 mutations carriers with breast cancer: a case series and literature review. Transl Breast Cancer Res.

[6] Boonen RA, Vreeswijk MP, van Attikum H (2020). Functional characterization of PALB2 variants of uncertain significance: toward cancer risk and therapy response prediction. Front Mol Biosci.

[7] Dillon KM, Bekele RT, Sztupinszki Z (2022). PALB2 or BARD1 loss confers homologous recombination deficiency and PARP inhibitor sensitivity in prostate cancer. NPJ Precis Oncol.

[8] Samad MA, Ahmad I, Khan MR (2025). Breast cancer: molecular pathogenesis and targeted therapy. MedComm (2020).

[9] Tischkowitz M, Balmaña J, Foulkes WD (2021). Management of individuals with germline variants in PALB2: a clinical practice resource of the American College of Medical Genetics and Genomics (ACMG). Genet Med.

[10] (2026). NCCN Clinical Practice Guidelines in Oncology (NCCN Guidelines®) for Genetic/Familial High-Risk Assessment: Breast, Ovarian, Pancreatic, and Prostate. Referenced with permission from the NCCN Clinical Practice Guidelines in Oncology (NCCN Guidelines®) for Genetic/Familial High-Risk Assessment: Breast, Ovarian, Pancreatic, and Prostate Version 3.2026. © National Comprehensive Cancer Network, Inc. 2026. All rights reserved. Accessed July 10, 2026. To view the most recent and complete version of the guideline, go online to NCCN.org. NCCN makes no warranties of any kind whatsoever regarding their content, use or application and disclaims any responsibility for their application or use in any way.

[11] Lowry KP, Geuzinge HA, Stout NK (2022). Breast cancer screening strategies for women with ATM, CHEK2, and PALB2 pathogenic variants: a comparative modeling analysis. JAMA Oncol.

[12] Crowley F, Gandhi S, Rudshteyn M, Sehmbhi M, Cohen DJ (2023). Adherence to NCCN genetic testing guidelines in pancreatic cancer and impact on treatment. Oncologist.

[13] Southey MC, Goldgar DE, Winqvist R (2016). PALB2, CHEK2 and ATM rare variants and cancer risk: data from COGS. J Med Genet.

[14] Tischkowitz M, Xia B (2010). PALB2/FANCN: recombining cancer and Fanconi anemia. Cancer Res.

[15] Vogt A, Schmid S, Heinimann K, Frick H, Herrmann C, Cerny T, Omlin A (2017). Multiple primary tumours: challenges and approaches, a review. ESMO Open.

